# Health System and Beneficiary Costs Associated With Intensive End-of-Life Medical Services

**DOI:** 10.1001/jamanetworkopen.2019.12161

**Published:** 2019-09-27

**Authors:** Risha Gidwani-Marszowski, Steven M. Asch, Vincent Mor, Todd H. Wagner, Katherine Faricy-Anderson, Samantha Illarmo, Gary Hsin, Manali I. Patel, Kavitha Ramchandran, Karl A. Lorenz, Jack Needleman

**Affiliations:** 1 Health Economics Resource Center, VA Palo Alto Health Care System, Menlo Park, California; 2 Center for Innovation to Implementation, VA Palo Alto Health Care System, Menlo Park, California; 3 Division of Primary Care and Population Health, Stanford University, Stanford, California; 4 Department of Health Services, Policy, and Practice, Brown University School of Public Health, Providence, Rhode Island; 5 Providence VA Medical Center, Providence, Rhode Island; 6 Department of Surgery, Stanford University, Stanford, California; 7 Alpert Medical School, Brown University, Providence, Rhode Island; 8 VA Palo Alto Health Care System, Palo Alto, California; 9 Division of Medical Oncology, Stanford University, Stanford, California; 10 Center for Health Policy/Center for Primary Care and Outcomes Research, Stanford University, Stanford, California; 11 Department of Health Policy and Management, UCLA Fielding School of Public Health, University of California, Los Angeles

## Abstract

**Importance:**

Despite recommendations to reduce intensive medical treatment at the end of life, many patients with cancer continue to receive such services.

**Objective:**

To quantify expected beneficiary and health system costs incurred in association with receipt of intensive medical services in the last month of life.

**Design, Setting, and Participants:**

This retrospective cohort study used data collected nationally from Medicare and the Veterans Health Administration for care provided in fiscal years 2010 to 2014. Participants were 48 937 adults aged 66 years or older who died of solid tumor and were continuously enrolled in fee-for-service Medicare and the Veterans Health Administration in the 12 months prior to death. The data were analyzed from February to August 2019.

**Exposures:**

American Society of Clinical Oncology metrics regarding medically intensive services provided in the last month of life, including hospital stay, intensive care unit stay, chemotherapy, 2 or more emergency department visits, or hospice for 3 or fewer days.

**Main Outcomes and Measures:**

Costs in the last month of life associated with receipt of intensive medical services were evaluated for both beneficiaries and the health system. Costs were estimated from generalized linear models, adjusting for patient demographics and comorbidities and conditioning on geographic region.

**Results:**

Of 48 937 veterans who received care through the Veterans Health Administration and Medicare, most were white (90.8%) and male (98.9%). More than half (58.9%) received at least 1 medically intensive service in the last month of life. Patients who received no medically intensive service generated a mean (SD) health system cost of $7660 ($1793), whereas patients who received 1 or more medically intensive services generated a mean (SD) health system cost of $23 612 ($5528); thus, the additional financial consequence to the health care system for medically intensive services was $15 952 (95% CI, $15 676-$16 206; *P* < .001). The biggest contributor to these differences was $21 093 (95% CI, $20 364-$21 689) for intensive care unit stay, while the smallest contributor was $3460 (95% CI, $2927-$3880) for chemotherapy. Mean (SD) expected beneficiary costs for the last month of life were $133 ($50) for patients with no medically intensive service and $1257 ($408) for patients with at least 1 medically intensive service (*P* < .001).

**Conclusions and Relevance:**

Given the low income of many elderly patients in the United States, the financial consequences of medically intensive services may be substantial. Costs of medically intensive services at the end of life, including patient financial consequences, should be considered by both physicians and families.

## Key Points

**Question:**

What is the cost associated with National Quality Forum–identified intensive medical services in the last month of life to beneficiaries and to the health care system?

**Findings:**

In this cohort study of 48 937 patients with cancer enrolled in Medicare and the Veterans Health Administration, those receiving no intensive service had a health system cost of $7660, whereas for the 59% of patients receiving 1 or more intensive services in the last month of life, the cost was $23 612. Expected beneficiary costs in the last month of life were $133 for patients with no intensive service and $1257 for patients with at least 1 intensive service.

**Meaning:**

Despite recommendations, more than half of patients with cancer receive intensive services at the end of life at a substantial cost to beneficiaries and the heath system.

## Teaser

To provide physicians with reference cost estimates, this cohort study quantifies beneficiary and health system costs incurred in association with receipt of intensive medical services in the last month of life among patients with advanced cancer.

## Introduction

A disproportionate share of medical spending is provided to patients in their last year of life.^1,2,3^ Much of that difference is no doubt because of unavoidable costs of serious illness.^4^ However, for patients with cancer, it is often possible to predict when intensive medical services have lost much of their potential benefit.^4^ For that reason, the National Academy of Medicine and the American Society of Clinical Oncology (ASCO) recommend a reduction in use of intensive medical services at the end of life, noting it is at odds with the focus on palliation and reduction in patient suffering that should characterize health care at this time.^5,6^

Despite such recommendations, patients continue to receive intensive medical services in the last month of life.^7,8^ Intensive services at the end of life are not linked to better outcomes,^9,10,11^ are associated with poorer patient quality of life,^12,13^ and are considered undesirable by many patients.^14,15^ An ancillary consequence of poor-quality end-of-life care is its cost, both to the health care system and to patients, who often bear nontrivial cost-sharing. Medical care is the leading cause of personal bankruptcy in the United States, and insurance is not sufficiently protective against patient financial consequences; most persons experiencing medical bankruptcy were insured at the time of their illness.^16^

Few studies have tried to quantify the financial consequences associated with these end-of-life intensive medical services. In the present study, we evaluate the additional costs incurred for patients who receive intensive medical services at the end of life, from both a health system and a beneficiary perspective. Although the medical community is no doubt aware that costs increase as use of health services increases, our goals in the present study are (1) to quantify the magnitude of that association, including for specific intensive medical services, and (2) to shed light on patient financial responsibility for medically intensive end-of-life services.

We focus the present study on patients dying of cancer, for 5 reasons. First, almost 40% of people in the United States will develop cancer at some point in their lives^17^; and cancer accounts for nearly 1 in 4 US deaths.^18^ Second, patient financial consequences for cancer care are especially high,^19^ with almost half of patients reporting cancer care–related financial strain,^20,21^ and many patients forgoing or discontinuing cancer treatment partly for financial reasons.^21,22^ Third, literature indicates death from solid tumor may be easier to prognosticate than death from other chronic illness.^4^ Fourth, the National Quality Forum (NQF)-endorsed ASCO guidelines for appropriate end-of-life care are premised on the assumption that death from cancer is able to be anticipated; therefore, there is a growing consensus that cancer-related death can often be forecast and that care decisions can be made based on that understanding. Fifth, in recognition of the high costs and limited health benefit of some cancer care, ASCO has recommended physicians discuss the value—the costs and likely outcomes—of treatment strategies with their patients. Yet physicians often have limited knowledge of the costs of care,^23,24^ making informed discussions challenging if not impossible. The present study quantifies the financial consequences of medically intensive end-of-life services and provides physicians with reference estimates that may be of use.

## Methods

Consistent with ASCO/NQF measures, we evaluated total costs of care in the last month of life for patients who did or who did not receive the following medically intensive services: 2 or more emergency department visits; chemotherapy; a hospital admission without an intensive care unit (ICU) stay; an ICU stay; or hospice for fewer than 3 days in the last month of life. We evaluated both oral and intravenous chemotherapy. Of note, ASCO/NQF include metrics for chemotherapy use in the last 14 days of life. Others have included chemotherapy use in the last 30 days of life to evaluate end-of-life care quality.^25,26,27^ Recognizing that the specific definition of “end of life” is subjective, and with a goal of being more inclusive, we similarly chose to evaluate chemotherapy in the last 30 days of life. This study followed the Strengthening the Reporting of Observational Studies in Epidemiology (STROBE) reporting guideline for cohort studies. This study was approved by and received a waiver of informed consent from the Stanford University Institutional Review Board because the research involved no more than minimal risk to the participants and the waiver would not adversely affect the rights and welfare of the participants.

### Study Population

Cohort members were veterans who died of cancer in fiscal years 2010 to 2014 and were identified using Surveillance, Epidemiology, and End Results–Medicare *International Statistical Classification of Diseases and Related Health Problems, Tenth Revision* (*ICD-10*) codes for underlying cause of death from National Death Index death certificate data.^28,29^ Underlying cause of death data have more than 89% sensitivity and more than 90% specificity for multiple solid tumor types.^30,31,32^ We made 2 adjustments to ASCO/NQF metrics to increase the likelihood that physicians were better able to anticipate their patients’ deaths: we limited the cohort to persons dying of solid tumor and to those who had an *ICD-9* code for cancer for at least 6 months in administrative data. These criteria have the effect of providing more conservative estimates of intensive medical services at the end of life than would strict ASCO/NQF criteria.

Cohort members were aged 66 years or older and were continuously enrolled in fee-for-service Medicare in the 12 months prior to death; the latter criterion allowed for full capture of use. Veterans aged 65 years or older are eligible to enroll in Medicare, and most dually enrolled veterans use both the Veterans Health Administration (VA) and Medicare for services.^33,34,35,36,37^ Medicare data were included in this analysis because other work indicates excluding Medicare substantially underestimates total costs of veteran care.^38^ We excluded patients who were also enrolled in Medicaid owing to the unavailability of those cost data; our cohort was limited to patients for whom we had complete capture of costs and utilization.

### Costs

The health system costs of intensive medical services in the VA were obtained from VA Managerial Cost Accounting data that we linked to inpatient, outpatient, and pharmacy administrative utilization data. Beneficiary costs in the VA are not directly available and were assigned using VA national office guidance, based on patients’ utilization of outpatient primary care, specialty care, and days of inpatient service use and enrollment priority.^39^ For example, patients who have an enrollment priority of 7 or 8 are eligible for copayments in VA. Patients in priority group 8 have the highest copayments; if these patients received inpatient care, their cost-sharing was $10 per day plus approximately $1200 for the first 90 days, and $600 for the next 90 days. Medicare health system and beneficiary costs and utilization were obtained from the MEDPAR, Outpatient, Carrier, Durable Medical Equipment, Hospice, Home Health and Part D administrative files. Beneficiary costs are a separate variable in the claims data present in these files. We also included care that was delivered in the community and paid for by the VA, known as Fee-Basis care. Taken together, these represent the entirety of costs incurred by patients in their last month of life. Medicare data are reimbursements for care provided, whereas VA data are cost estimates based on activity-based cost accounting. Patient costs in both systems are beneficiary expectations of payment, rather than patient copayments because the latter are not available in the Medicare or VA research data. They therefore represent the starting point for patient copayments, rather than final patient copayments; yet they remain the most comprehensive data available for research purposes. For simplicity, we refer to all as costs. Costs were inflation adjusted to 2014 dollars using the personal consumption expenditures index,^40^ as recommended by the Second Panel on Cost-Effectiveness in Health and Medicine.^41^

### Statistical Analysis

We estimated the association between receipt of medically intensive services and costs in the last month of life using a generalized linear model. A modified Park test and a Box-Cox regression recommended a gamma distribution with a log link function.^42^ Models were adjusted for age as a categorical variable, race, the cancer category representing the underlying cause of death, and Elixhauser comorbidities.^43^ Models were conditioned on geographic area (hospital referral region) to account for both geographic differences in the practice of end-of-life care^9^ and geographic variation in wages; this method allows for interpretation of the cost differences between medically intensive and nonmedically intensive services within geographic regions.

Models were used to estimate the additional costs associated with medically intensive services, with bias-corrected 95% CIs generated through bootstrapping with 1000 replications. We adjusted for multiple hypothesis testing by constraining the familywise error rate to no more than 0.05 (2-tailed level of significance) across the 5 outcomes studied.^44^ All analyses were conducted in Stata/MP, version 15.1 (StataCorp), from February to August 2019.

## Results

The study cohort consisted of 48 937 veterans who received care through the VA and Medicare. Our cohort was majority white (90.8%), male (98.9%), and had a mean (SD) of 8.8 (3.9) comorbidities (Table 1). Cohort members were most likely to die of lung and bronchus cancer (31.1%) or prostate cancer (20.8%). Cohort members had a cancer diagnosis for a median (interquartile range) of 34.6 (16.0-54.8) months before death. In unadjusted analyses, patients who received a medically intensive service had a longer time from cancer diagnosis to death than patients who did not (*P* < .001 using a Wilcoxon rank sum test) (Table 1).

**Table 1.** Demographic Characteristics and Comorbidities

Characteristic	No. (%) of Patients			*P* Value
	Total	Did Not Receive Medically Intensive Services	Received Medically Intensive Services	
Total, No.	48 937	20 102	28 835	
Age, y				
66 to <71	9870 (20.2)	3675 (18.3)	6195 (21.5)	<.001
71 to <76	7302 (14.9)	2736 (13.6)	4566 (15.8)	
76 to <81	9990 (20.4)	4015 (20.0)	5975 (20.7)	
81 to <86	10 421 (21.3)	4435 (22.1)	5986 (20.8)	
86 to <91	8391 (17.2)	3857 (19.2)	4534 (15.7)	
≥91	2963 (6.0)	1384 (6.9)	1579 (5.5)	
Male	48 378 (98.9)	28 557 (99.0)	19 821 (98.6)	<.001
Eligible for copayments in VA	15 283 (31.2)	6520 (32.4)	8763 (30.4)	<.001
Race				<.001
White	25 116 (51.3)	18 242 (90.8)	25 116 (87.1)	
Black	4365 (8.9)	1408 (7.0)	2957 (10.2)	
Asian/Hawaiian/Pacific Islander	437 (0.9)	148 (0.7)	289 (1.0)	
Missing	346 (0.7)	151 (0.8)	195 (0.7)	
Mixed	286 (0.6)	103 (0.5)	183 (0.6)	
Native American	145 (0.3)	50 (0.2)	95 (0.3)	
Rural status				
Highly urban	20 091 (41.0)	7811 (38.9)	12 280 (42.6)	<.001
Rural	21 608 (44.2)	9194 (45.7)	12 414 (43.0)	
Urban	7166 (14.6)	3067 (15.3)	4099 (14.2)	
Missing	72 (0.2)	30 (0.2)	42 (0.2)	
Underlying cause of death				
Bladder	2832 (5.8)	1121 (5.6)	1711 (5.9)	<.001
Brain and nervous system	655 (1.3)	309 (1.5)	346 (1.2)	
Colorectal	4158 (8.5)	1870 (9.3)	2288 (7.9)	
Gastroesophageal	2458 (5.0)	987 (4.9)	1471 (5.1)	
Head and neck	1491 (3.1)	517 (2.6)	974 (3.4)	
Hepatobiliary	1703 (3.5)	710 (3.5)	993 (3.4)	
Kidney	1499 (3.1)	663 (3.3)	836 (2.9)	
Lung and bronchus	15 241 (31.1)	6141 (30.6)	9100 (31.6)	
Melanoma	1040 (2.1)	490 (2.4)	550 (1.9)	
Other	5460 (11.2)	2040 (10.2)	3420 (11.9)	
Pancreas	2214 (4.6)	930 (4.6)	1284 (4.4)	
Prostate	10 186 (20.8)	4324 (21.5)	5862 (20.3)	
Comorbidities				
Chronic obstructive pulmonary disorder	25 008 (51.1)	9845 (49.0)	15 163 (52.6)	<.001
Renal disease	14 497 (29.6)	5536 (27.5)	8961 (31.1)	<.001
Diabetes without chronic complications	18 325 (37.5)	7066 (35.2)	11 259 (39.1)	<.001
Diabetes with chronic complications	6414 (13.1)	2406 (12.0)	4008 (13.9)	<.001
HIV/AIDS	81 (0.2)	21 (0.1)	60 (0.2)	.01
Peripheral vascular disease	15 367 (31.4)	6181 (30.8)	9186 (31.9)	.01
Moderate or severe liver disease	869 (1.8)	339 (1.7)	530 (1.8)	.21
Mild liver disease	7993 (16.3)	3226 (16.0)	4767 (16.5)	.15
Hemiplegia or paraplegia	1666 (3.4)	682 (3.4)	984 (3.4)	.91
Acute myocardial infarction	6826 (14.0)	2653 (13.2)	4173 (14.5)	<.001
Cerebrovascular disease	12 632 (25.8)	5133 (25.5)	7499 (26.0)	.24
Dementia	3297 (6.7)	1528 (7.6)	1769 (6.1)	<.001
Rheumatologic disease	1586 (3.2)	593 (3.0)	993 (3.4)	.01
Peptic ulcer disease	2369 (4.8)	882 (4.4)	1487 (5.2)	<.001
Congestive heart failure	13 876 (28.4)	5296 (26.4)	8580 (29.8)	<.001
Months between first cancer diagnosis and death, median (IQR)	34.6 (16.0-54.8)	33.3 (15.7-54.3)	35.6 (16.3-55.1)	<.001

Abbreviations: IQR, interquartile range; VA, Veterans Health Administration.

More than half of the cohort (58.9%) received at least 1 medically intensive service in the last month of life (Table 2). Patients who received medically intensive services were significantly more likely to have comorbidities, although the difference was small (Table 1). The most frequently occurring intensive service was insufficient hospice exposure (36.6%), followed by a hospital stay without an ICU admission (30.3%); the least frequently occurring intensive service was chemotherapy (11.0%) (Table 2). Patients were most likely to receive 1 (28.6%) or 2 (22.1%) intensive services in the last month of life; few patients had 3 or more intensive services.

**Table 2.** Receipt of Medically Intensive Services in the Last 30 Days of Life

Medically Intensive Service	No. (%) of Patients
Type	
Any	28 835 (58.9)
>2 ED visits	6159 (12.6)
Chemotherapy	5392 (11.0)
Hospital admission without ICU stay	14 810 (30.3)
Hospital admission with ICU stay	7038 (14.4)
Hospice for ≤3 d	17 886 (36.6)
No.	
0	20.102 (41.4)
1	12 527 (28.6)
2	10 826 (22.1)
3	4822 (9.9)
4	660 (1.4)
5	0

Abbreviations: ED, emergency department; ICU, intensive care unit.

Receipt of medically intensive services was associated with higher costs of care, for both the health system and the beneficiary. Patients with at least 1 medically intensive service had $15 952 (95% bias-corrected CI, $15 676-$16 206; *P* < .001) greater health system costs in the last month of life compared with patients who did not have any medically intensive service (mean [SD], $23 612 [$5528] vs $7660 [$1793]). They also had $1123 (95% CI, $1115-$1143) greater expected beneficiary costs (Table 3).

**Table 3.** Estimated Costs of Medically Intensive Care for the Health System and the Beneficiary in the Last Month of Life

Service	Cost for No Medically Intensive Service, $	Cost for Receipt of Medically Intensive Service, $^a^
Any intensive service		
Health system	7660	23 612
Patient	133	1257
>2 ED visits		
Health system	15 868	27 007
Patient	675	1553
Chemotherapy		
Health system	16 919	20 379
Patient	680	1621
Hospital admission without ICU stay		
Health system	14 699	23 289
Patient	557	1327
Hospital admission with ICU stay		
Health system	14 142	35 235
Patient	608	1830
Hospice for ≤3 d		
Health system	12 303	25 438
Patient	488	1300

Abbreviations: ED, emergency department; ICU, intensive care unit.

^a^ All differences between no medically intensive service and receipt of such service are statistically significant after adjusting for multiple hypothesis testing (all *P* < .001).

**Figure 1.**
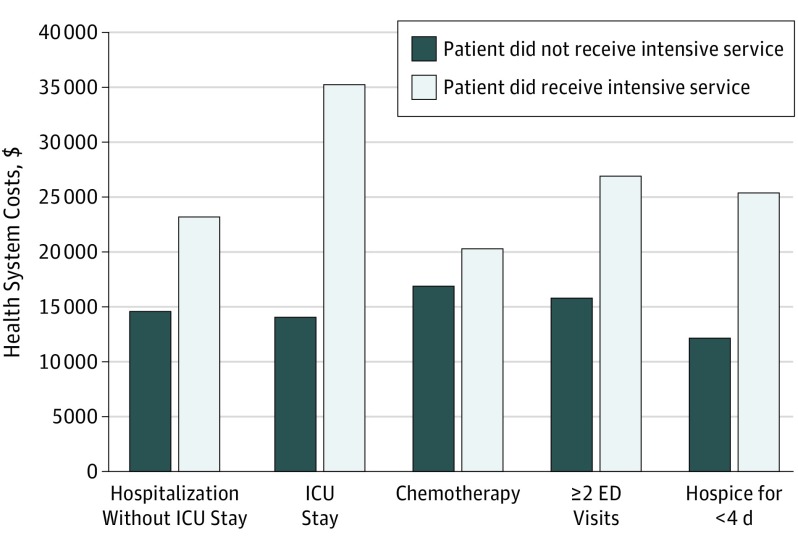
Health System Costs in the Last Month of Life due to Medically Intensive Services ED indicates emergency department; ICU, intensive care unit.

**Figure 2.**
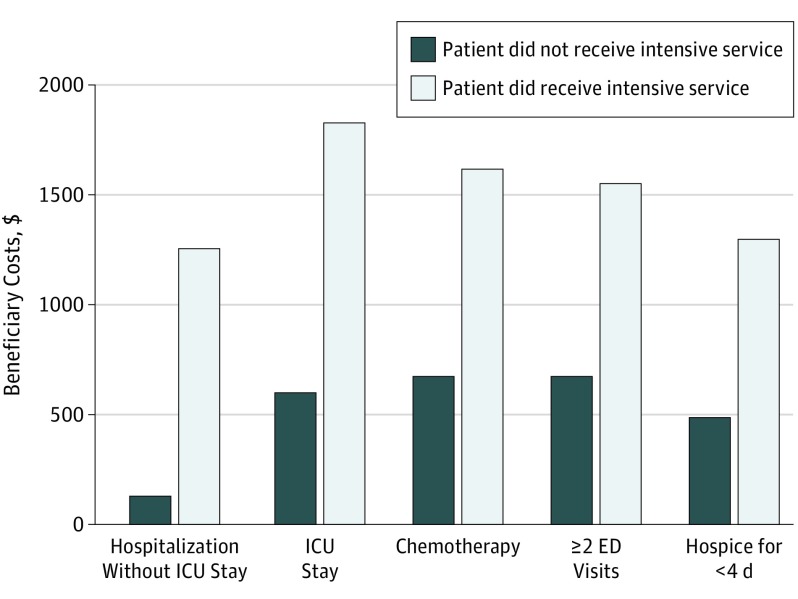
Beneficiary Costs in the Last Month of Life Due to Medically Intensive Services ED indicates emergency department; ICU, intensive care unit.

Costs varied substantially by type of intensive service provided, with ICU stays associated with highest additional financial consequence and chemotherapy associated with lowest additional financial consequence for the health system (Figure 1 and Figure 2). Patients with an ICU stay in the last month of life had $21 093 (95% CI, $20 364-$21 689) higher health system costs and $1222 (95% CI, $1178-$1238) higher expected beneficiary costs than those who did not have an ICU stay. Patients with a non-ICU hospital stay had $8590 (95% CI, $8224-$8772) higher health system costs and $771 (95% CI, $749-$778) higher expected beneficiary costs than those who did not have a non-ICU hospital stay. Patients with 2 or more emergency department visits in the last month of life had $11 140 (95% CI, $10 623-$11 495) higher health system costs and $879 (95% CI, $853-$901) higher expected beneficiary costs. Patients who received 3 or fewer days of hospice had $13 134 (95% CI, $12 713-$13 501) higher health system costs and $811 (95% CI, $778-$825) higher expected beneficiary costs than those who received hospice for the greater (recommended) number of days. Patients who received chemotherapy had $3460 (95% CI, $2927-$3880) higher health system costs and $942 (95% CI, $888-$969) higher expected beneficiary costs than those who did not receive any chemotherapy in the last month of life.

The total estimated mean (SD) health care system costs for patients with intensive services were as follows: $23 612 ($5528) for any intensive service; $35 235 ($10 067) for hospital admission with ICU stay; $27 007 ($8629) for 2 or more emergency department visits; $25 438 ($6729) for hospice lasting 3 or fewer days; $23 289 ($7495) for hospital admission without ICU stay; and $20 379 ($6646) for chemotherapy.

The total estimated mean (SD) expected beneficiary costs for patients with intensive services were as follows: $1257 ($468) for any intensive service; $1830 ($652) for hospital admission with ICU stay; $1621 ($591) for chemotherapy; $1553 ($585) for 2 or more emergency department visits; $1327 ($515) for hospital admission without ICU stay; and $1300 ($474) for hospice lasting 3 or fewer days (Table 3).

In models evaluating the association between the number of intensive services provided and costs, (mean [SD]) health care systems costs were similar for patients with 2 ($27 979 [$6432]), 3 ($28 641 [$6589]), or 4 ($28 854 [$6638]) intensive services (all *P* > .05). The greatest differences in health system costs were between 0 and 1 intensive services (mean [SD], $7617 [$1752] vs $17 814 [$4099]); *P* < .001). There were also significant differences in costs between 2 services vs 0 services and 2 services vs 1 service (both *P* < .001). However, the differences in health system costs between 2 services and 3 or 4 services was not significant (*P* = .13 for 2 vs 3; *P* = .39 for 2 vs 4; *P* = .85 for 3 vs 4). By contrast, expected beneficiary costs increased significantly with each intensive service provided (mean [SD]: $133 [50] for no intensive services; $820 [$309] for 1 intensive service; $1463 [$552] for 2 intensive services; $1830 [$691] for 3 intensive services; and $2230 [$842] for 4 intensive services). For expected beneficiary costs, all *P* values were .01 or less for each number of service vs other numbers of services (2 services vs 0 services, 2 services vs 1 service, etc).

## Discussion

Despite recommendations to the contrary, more than half of cancer decedents receive medically intensive services in the last month of life. We found the costs associated with nonrecommended intensive services added a mean of almost $16 000 to health care system costs and more than $1100 to expected beneficiary costs in the last month of life alone, bringing total spending in the last month of life to a mean of $24 000 for the health system and $1300 for the beneficiary. Although it is not surprising that intensive medical services cost more, quantifying the magnitude of these costs can help spur efforts to reduce it.

Prior studies have estimated overall health system costs in the last 6 months of life for Medicare beneficiaries to range from means of $41 712 to $74 212^45,46^ and medians around $22 000.^47,48^ To our knowledge, no other study has quantified the additional costs associated with intensive medical services at the end of life, and there is no other published evaluation of beneficiary expectations of payment or patient costs associated with intensive medical services at the end of life. In this work, we found ICU stays were associated with the highest excess financial consequence to the health system, of more than $21 000. Chemotherapy was associated with the lowest excess financial consequence to the health system of approximately $3500. The low excess financial consequence of chemotherapy is partially because patients not receiving chemotherapy were still receiving other intensive services in the last month of life, contributing to their total costs.

Many factors influence the patient-clinician decision to pursue medically intensive services. These may include patient desire for such care, clinician belief that it will provide significant medical or palliative benefit, patient denial of limited prognosis, or miscommunication about prognosis. However, when querying patients about what they would hypothetically prefer, they often assert that intensive services at the end of life are undesirable.^49,50,51^ Evidence also indicates that patients and clinicians may not always share the same understanding about prognosis; in 1 large sample study, more than two-thirds of patients with metastatic solid tumor were unaware that their chemotherapy had no curative intent.^52^ Thus, miscommunication or misunderstanding of prognosis is of particular concern.

Ideally, patient-clinician decisions to pursue medically intensive services should involve discussions of the likelihood of benefit, risks, and side effects, including potential financial consequences, of these interventions. The present study provides reference cost estimates that may help inform those discussions. The present analysis indicates that patients experience approximately $1250 out-of-pocket health costs in the last month of life due to medically intensive services. To place this number in context, the median annual household income of a Medicare beneficiary in 2014, the last year of this analysis, was $24 150,^53^ or $2013 a month. Using these figures, expected beneficiary spending on medical services that have a low likelihood of helping them and could harm them may represent 62% of the household income of the typical Medicare enrollee in the last month of his life. Indeed, analyses of Medicare-only beneficiaries find that beneficiaries with a new cancer diagnosis have out-of-pocket costs that are a mean of 24% of their household income.^54^ The present study indicates that as cancer progresses, expected beneficiary responsibility for intensive medical services represents a higher proportion of household income, rising to almost two-thirds of household income in the last month of life. This study also identified costs in the last month of life only, and almost 90% of our cohort received no chemotherapy in this time frame. Thus, analyses looking farther back from death would yield much higher estimates of drug costs. This, coupled with the high cost of chemotherapies and immunotherapies that have come to market after fiscal year 2014 (the last year of our data),^55,56^ indicate that the financial consequences due to cancer care will only grow.

The purchasing of medical care, including chemotherapy and end-of-life care, is largely guided by physicians; recent work finds that physician beliefs, rather than patient beliefs, explain much of oft-noted geographic variation in end-of-life spending.^57^ Thus, physicians have a strong opportunity to limit medically intensive interventions at the end of life in ways that can avoid patient financial consequences. For example, early goals-of-care conversations with patients may help to ensure that patients receive the care they want and need. The present study also highlighted that given the low likelihood of benefit and the potential for financial consequences, as well as patient concerns about the cost of cancer care,^21,22^ it may be worthwhile for physicians to discuss this openly with patients before proceeding with medically intensive interventions.

### Limitations

The present study was subject to certain limitations. First, this study was a retrospective evaluation of cancer decedents, and although we examined care for patients provided in the month prior to death, patients and their treating oncologists may have been less certain that the patients were in their final month of life. However, oncologists regularly use many clinical indicators to help prognosticate life expectancy, including understanding the expected trajectory of a specific solid tumor as it metastasizes, recognizing the rapidity of a patient’s personal disease progression, and identifying symptoms that death is becoming imminent. Several tools have been developed to aid in this prognostication.^58,59,60^ In fact, ASCO has created specific metrics regarding care in the last 30 days of life, indicating the importance the oncology community places on attempting to anticipate when patients are approaching death.

Second, as noted above, this study evaluates the beneficiary expectation of payment, rather than the actual costs paid by patients, as the latter are not available in Medicare and VA research databases. On the Medicare side, the majority of enrollees in traditional Medicare have supplemental insurance,^61^ often referred to as Medigap insurance, that covers much of their cost-sharing. However, while supplemental insurance reduces patients’ out-of-pocket costs, it does not eliminate them. Survey analyses found 1-year out-of-pocket payments by patients with cancer and Medigap insurance were $5670 vs $8115 for patients without Medigap insurance.^54^ When factoring in premiums for Medigap insurance and Part D plans (the former conservatively estimated to be $1440 per year),^62^ the differences in patient total cost-sharing between patients with cancer and with or without Medigap insurance narrows further (estimated to be $8115 vs $7110 for patients with and without Medigap, not including the cost of Part D premiums). Part D coverage for oral medications, including oral chemotherapy, also does not fully protect patients from cost-sharing. Even after closure of the Medicare Part D “coverage gap,” Part D patient out-of-pocket costs for oral chemotherapy are estimated to be $5663 for an average course of treatment.^63^ Taken together, our results as well as published information about Medicare cost-sharing indicate substantial patient financial responsibility for care provided in the last month of life, regardless of supplemental insurance status. In addition, the premiums patients pay for supplemental insurance are heavily influenced by Medigap expenditures for previous years. Finally, because VA care is not eligible for supplemental insurance, our VA-measured costs are a reasonable approximation or actual out-of-pocket payments, as is the cost-sharing associated with Medicare Part D services. Furthermore, 20% of the eligible Medicare population does not have supplemental insurance^61^; beneficiary payments do equal out-of-pocket spending for that group, and given that their income distribution of that population tends to skew low,^61^ the beneficiary costs we present are particularly relevant for that group. Given all of these factors, understanding the amount beneficiaries are expected to pay or must have reimbursed through secondary insurance they purchase remains important yet understudied.

Third, our work combined VA and Medicare data for a cohort of veterans. This produces conservative estimates of expected beneficiary responsibility for care because our beneficiary cost data include both VA and Medicare expenses, and patient cost-sharing in VA is nominal. Many veterans pay no copayments. In our cohort, approximately one-third of veterans were eligible for copayments in VA (although all were eligible for copayments in Medicare). Even those veterans who are eligible for cost-sharing in the VA have greater financial protection than veterans seeking care through Medicare. For example, veterans who are eligible for VA copayments pay $15 per primary care visit, and $50 per specialty care visit. By contrast, Medicare charges a 20% coinsurance for outpatient services, including for infused chemotherapies. In addition, unlike Medicare, VA has a drug formulary and negotiates drug prices. Thus, an analysis evaluating a nonveteran cohort would yield much higher figures for expected beneficiary costs in the last month of life. However, although the use of a veteran cohort limits the generalizability of our findings, it has the advantage of ensuring findings regarding intensive services are not driven by the fee-for-service incentives for overuse of services present in Medicare. Indeed, our results point to the need for further efforts to improve the value of end-of-life care in both integrated and fee-for-service environments.

## Conclusions

The present study is, to our knowledge, the first analysis of both health system costs and beneficiary costs associated with medically intensive end-of-life care. Our results indicated that receipt of medically intensive services in the last month of life was associated with substantial costs, a nontrivial portion of which was borne by beneficiaries and their decedents. Efforts to more appropriately use medical care may benefit by considering beneficiary financial consequences as well as health system costs associated with intensive medical interventions. Our results provide further support for the consideration of value in cancer care.
